# EMT-Associated Heterogeneity in Circulating Tumor Cells: Sticky Friends on the Road to Metastasis

**DOI:** 10.3390/cancers12061632

**Published:** 2020-06-19

**Authors:** Anthony Genna, Aline M. Vanwynsberghe, Amélie V. Villard, Charles Pottier, Julien Ancel, Myriam Polette, Christine Gilles

**Affiliations:** 1 GIGA-Cancer, Laboratory of Tumor and Development Biology, CHU Sart-Tilman, University of Liège, Pathology Tower, 4000 Liège, Belgium; anthony.genna@uliege.be (A.G.); aline.vanwynsberghe@uliege.be (A.M.V.); amelie.villard@uliege.be (A.V.V.); charles.pottier@chuliege.be (C.P.); 2 Department of Medical Oncology, University Hospital of Liège, 4000 Liège, Belgium; 3 CHU (Centre Hopitalier Universitaire) de Reims, Hôpital Maison Blanche, Service de Pneumologie, 51092 Reims, France; jancel@chu-reims.fr; 4 INSERM, UMR (Unité Mixte de Recherche)-S1250, SFR CAP-SANTE, Université de Reims Champagne-Ardenne, 51097 Reims, France; myriam.polette@univ-reims.fr; 5 CHU de Reims, Hôpital Maison Blanche, Laboratoire de Pathologie, 51092 Reims, France

**Keywords:** EMT, CTC, early metastasis, heterogeneity, coagulation

## Abstract

Epithelial–mesenchymal transitions (EMTs) generate hybrid phenotypes with an enhanced ability to adapt to diverse microenvironments encountered during the metastatic spread. Accordingly, EMTs play a crucial role in the biology of circulating tumor cells (CTCs) and contribute to their heterogeneity. Here, we review major EMT-driven properties that may help hybrid Epithelial/Mesenchymal CTCs to survive in the bloodstream and accomplish early phases of metastatic colonization. We then discuss how interrogating EMT in CTCs as a companion biomarker could help refine cancer patient management, further supporting the relevance of CTCs in personalized medicine.

## 1. General Background

Circulating tumor cells (CTCs) contain the physical entities that cause metastases and therefore hold a special place in the era of liquid biopsies [1,2,3,4]. Although tumor biopsy is still the gold standard for cancer diagnosis of solid tumors, it is an invasive act both at the primary and metastatic sites, and it represents a snapshot during the progression of the disease. Analyzing CTCs through successive liquid biopsies may thus provide important additional clinical information.

The first observation of CTCs actually dates back to 1869, when Thomas Ashworth reported the presence of cells “with similar characteristics than those of the primary tumor” in the blood of a cancer patient [5]. Enumeration and characterization of CTCs may improve precision oncology through predicting metastases, monitoring recurrence, guiding treatment decisions and patient stratification, and assessing therapeutic efficacy [6,7].

Progressively understanding that CTCs represent a very heterogeneous population has urged researchers to examine epithelial–mesenchymal transitions (EMTs) and to characterize metastatic founders within the CTC population.

Nevertheless, although the clinical validity of analyzing CTCs as prognostic and predictive biomarkers is currently supported by many studies, they have still not been examined in clinical practice [8]. The technical challenge behind the isolation of these extremely rare cells may contribute to hampering their exploitation in the clinic [9,10,11,12].

## 2. CTC Enrichment, Identification, and Isolation Techniques

**Figure 1:**
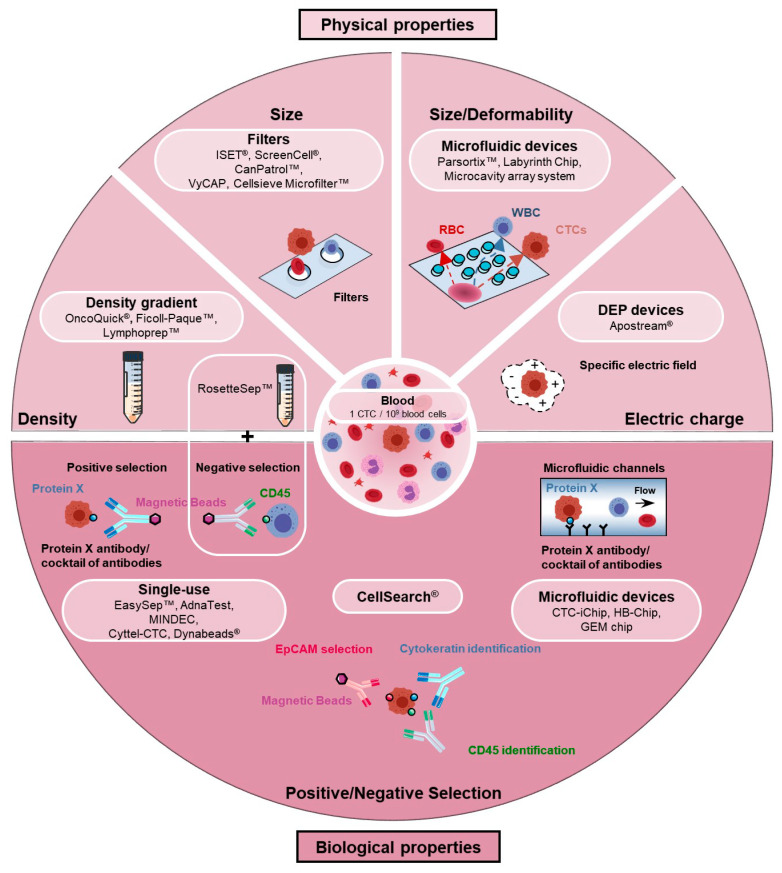
Circulating tumor cell (CTC) enrichment techniques. Current devices/methods used to enrich and isolate CTCs exploit biological or biophysical properties to differentiate CTCs from blood cells. CTC enrichment methods based on biological properties take advantage of biological markers differentially expressed in CTCs and blood cells. Positive selection of CTCs and/or depletion (negative selection) of blood cells may thus be achieved using a specific antibody (such as EpCAM) or cocktails of antibodies. Immunomagnetic separation is used in many systems and kits (CellSearch^®^, EpCAM PlusCellect^TM^ Kit, EasySep^TM^ human EpCAM positive kit, EpCAM positive CELLection^TM^ beads or AdnaTest) but an abundance of microfluidic devices (CTC-Chip, CTC-iChip, HB-Chip or GEM Chip) has also been developed. CTC enrichment methods based on physical characteristics use the following criteria to separate tumor cells from blood cells: Size (filter-based methods: ISET^®^, ScreenCell^®^, VyCap, CanPatrol^TM^), deformability/size (microfluidic devices: Parsortix^TM^, Labyrinth chip, microcavity array system), density (ficoll-type density gradients: OncoQuick^®^, Ficoll-Paque^TM^, Lymphoprep^TM^ or RosetteSep^TM^ that combines an immune-depletion of white blood cells), and electric charge (Apostream^®^).

CTC enrichment/detection/isolation methods have been reviewed elsewhere [9,10,11,12]. We here recapitulate the general principles behind these techniques (Figure 1). Very schematically, one may distinguish enrichment systems based on biological characteristics of CTCs and those based on their physical properties. Methods combining both approaches are also frequently used.

Enrichment techniques based on CTC biological properties assume that CTCs express or do not express specific markers that can be used to separate them from normal cells. This is achieved either by positively selecting cells expressing a specific marker or a combination of markers, or/and depleting populations of blood cells (negative selection). The CellSearch^®^ is the only system that has been approved by FDA for CTC enumeration in metastatic breast, prostate, and colorectal cancer patients [13,14,15,16]. Based on an EpCAM immunomagnetic enrichment and a keratin^+^/CD45^−^ identification, it is still considered a gold standard in CTC research. Aside the CellSearch^®^, other EpCAM-based immunomagnetic enrichment kits are also commonly used. It was nevertheless rapidly appraised that EpCAM is not a universal CTC marker, and that EpCAM-negative CTC populations may encompass metastatic precursors that will not be detected by such methods, particularly those derived from EMTs [17,18]. Several studies have indeed reported that EMTs decrease EpCAM levels in many, although not all, examined cellular backgrounds [17]. Systems using cocktails of antibodies have thus been developed to enrich more CTC populations. Conversely, negative selection approaches have also been developed. If many of these systems use immunomagnetic sorting, microfluidic-based enrichment technologies have also emerged, in which different supports coated with specific antibodies are precisely disposed in the flow so as to favor cell–antibody interactions [19,20,21,22,23,24,25,26,27,28,29,30]. Interestingly, aptamers are gaining major interest as an alternative to antibodies in positive selection-based CTC enrichment, and have for instance been exploited in magnetic bead separation assays or in microfluidic devices [31,32]. Aptamers are short DNA/RNA molecules with unique tertiary structures that bind specific targets, including proteins, with high specificity and affinity, and that may additionally be easily removed from their targets. Aptamers against EpCAM, EGFR, or MUC1 have for example been successfully generated.

As mentioned above, a general drawback of these techniques based on biological characteristics is their inability to enrich CTC subsets that do not express the examined biological markers. To circumvent this problem and improve the capture of EMT^+^ CTCs, and to enable the isolation of label-free CTCs that may facilitate downstream applications, an abundance of enrichment devices using biophysical parameters have been engineered. Size, density, deformability, and electric charge are most commonly at the basis of these assays. Thus density-based approaches often involve a centrifuged Ficoll-type density gradient [33,34,35,36,37]. Several filter-based assays exploit the knowledge that most hematopoietic cells are smaller (<10 µm) than CTCs (>10 µm) [38,39,40,41,42,43,44,45]. A profusion of recently conceived microfluidic devices also uses the size and/or the deformability parameters [46,47,48,49,50,51,52,53,54,55,56,57,58]. Finally, CTC isolation devices, many also being microfluidic-based [59,60,61,62,63], use dielectrophoresis (DEP) to differentiate tumor cells from normal cells by their electric charges [64].

The prominent place progressively taken by microfluidics in the CTC field within the last decade is worth highlighting [19,20,21,22,23]. Microfluidics has indeed emerged as an innovative approach to directly process whole blood, to use small amounts of reagents, and to reduce cost. By controlling the flow rate and the design of the chip in a coordinated manner, the capture efficiency and purity may eventually be improved. Microfluidic chips may also be optimized to particularly favor the isolation of viable label-free CTCs and to facilitate various specific downstream applications. An impressive number of microfluidic devices have been/are being designed to dispose obstacles (channels, pillars, labyrinths, spirals, weirs, layers, filters, ratchets) in flow chambers so as to efficiently separate CTCs from normal cells contained in the blood [28,50,56,57,58,65,66,67].

Thus far, spiking experiments, the gold standard for evaluating the efficiency of CTC isolation devices, have revealed important differences in capture efficiency among all these biological or biophysical property-based devices. If these discrepancies are certainly in part inherent to the technology used in each device, it also appears that the phenotype and the specific characteristics of tumor cells influence the recovery rate. CTC enrichment systems are thus still being optimized to increase isolation efficiency and purity but also to broaden the capture to different CTC phenotypes.

The engineering of a single CTC isolation device that addresses all the technical challenges currently seems unlikely. It may be more realistic to select an appropriate CTC isolation device with respect to the downstream application envisaged and the clinical question asked.

## 3. Epithelial–Mesenchymal Transitions: Impact on CTC Phenotypic Heterogeneity

EMTs have long been known as crucial actors in metastasis. The examination of EMT actors in CTCs has thus logically gained rapidly growing interest in the past decade [68,69,70,71,72,73,74].

The generally accepted view [75,76,77,78,79,80,81] is that EMTs generate various hybrid phenotypes along the epithelial (E) to mesenchymal (M) differentiation axis, thereby contributing importantly to tumor heterogeneity. If most epithelial and mesenchymal states are believed to harbor limited metastatic potential, certain E/M hybrid phenotypes are considered to harbor high degree of epithelial–mesenchymal plasticity (EMP), enabling them to undergo timely and spatially regulated dynamic and reversible interconversions within a “plasticity window”. These phenotypical adaptations are crucial for tumor cells to survive/develop in the different microenvironments encountered during the metastatic spread. After an eventual period of dormancy, a switch towards more epithelial proliferative states (mesenchymal–epithelial transitions, METs) is further suspected to occur during metastatic outgrowth. EMTs would therefore rather be involved in the initial steps of the metastatic spread: entry in the circulation, survival in the bloodstream, arrest on the vasculature, and early phases of metastatic niching [68,75,76,77,78,79,80,81,82,83]. Whether the same hybrid tumor cell is able to overcome all obstacles of the metastatic cascade through phenotypic adaptations or whether further genetic alterations occur during the metastatic cascade that empower some tumor cells to form metastases is still a subject of debate. Cooperative processes between different phenotypes may also occur, by which EMT-shifted cells would help more epithelial phenotypes (with higher competence for metastatic outgrowth) to gain and survive in the circulation, and niche in secondary organs [84,85].

Adding to the heterogeneity generated by this phenotypic plasticity occurring throughout the metastatic spread, EMTs are molecularly complex, diverse, and context-dependent.

Several EMT-associated genes have nevertheless been commonly examined in CTC studies. Among EMT target genes frequently examined in CTCs is certainly vimentin. This mesenchymal type III intermediate filament is considered a canonical marker of EMT and has been extensively examined both in tumors and CTCs over the years. More than being a marker, vimentin has also been functionally implicated in pro-metastatic functions including tumor cell migration or CTC survival [68]. In addition, EMTs are known to modulate the expression of several epithelial adhesion molecules, consequently altering cell–cell interactions. A cadherin switch characterized by a decrease of E-cadherin expression and an enhanced expression of N-cadherin has accordingly been associated with EMTs [86], and both molecules are frequently analyzed in CTCs. The adhesion molecule EpCAM, which, as discussed underneath, has been used in pioneer studies to enrich CTCs, has also been identified as an EMT target gene examined in many CTC studies [17]. EMT core transcription factors which finely regulate EMT target genes [87,88,89] are also commonly assessed in CTCs, particularly those of the ZEB (ZEB1 and ZEB2) and Snail (Snail and Slug) families, and Twist. Several membrane receptor and Receptor Tyrosine Kinase (RTK) signaling pathways, transmitting signals from the microenvironment, are key regulators of EMTs [90] and have been analyzed in many CTC studies. These include TGF-β, EGFR, c-Met, Notch, and Wnt pathways. The availability of targeted drugs against specific RTKs or their signaling molecules has also stimulated this axis of investigations. A growing interest in the EMT–associated RTK Axl, for which commercial inhibitors are being assessed in clinical trials [91,92,93], may be underlined. Although herein we will not debate the exact nature of the relationships linking EMT and Cancer-Stem Cells (CSCs) [94,95], it is important to note that EMT induces the expression of stem cell attributes [96,97] and that certain EMT and CSC markers are often coexpressed in tumor cells. Stem cell markers including CD44 (considered a marker of both EMT and CSC [98,99]), ALDH1 or CD133 (promin 1) are thus frequently appraised in CTCs, often in association with canonical EMT markers. As we detail underneath, many of these EMT-associated molecules analyzed in CTCs functionally impact CTC survival and metastatic potential.

As shown in Table 1 (for studies published before 2016, please refer to Table 1 in [68]), a profusion of studies has revealed EMT-associated heterogeneity in the CTC population, and the presence of CTCs encompassing hybrid E/M phenotypes in most types of epithelial cancers.

## 4. Epithelial–Mesenchymal Transitions: Impact on Metastatic Competence

**Figure 2:**
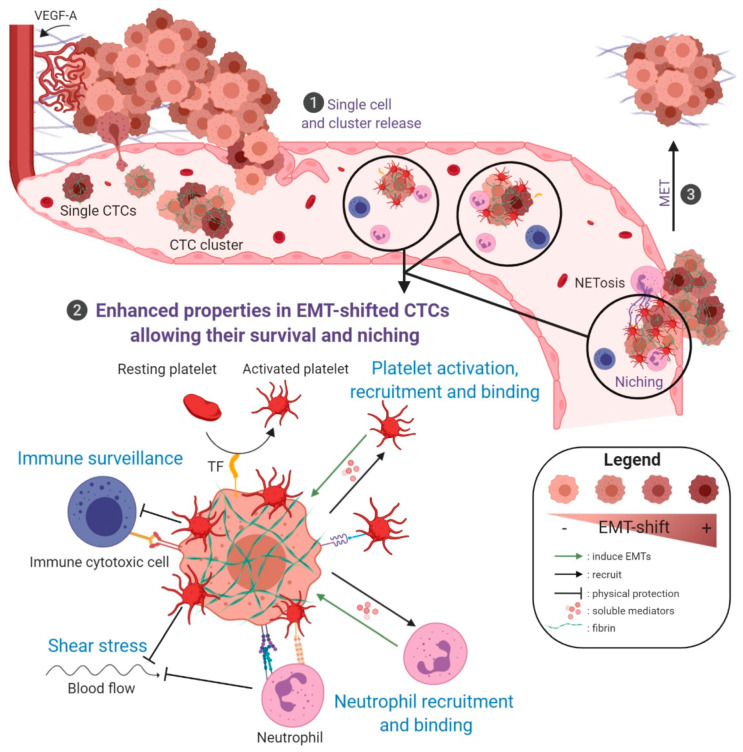
Schematic representation of EMT-associated mechanisms supporting CTC survival and early metastasis. ❶ CTCs are liberated in the bloodstream through EMT-associated mechanisms (single cell or collective migration/intravasation) or passive processes (detachment of isolated tumor cells or clumps through corrupted vessels). ❷ Some single or clustered CTCs will eventually survive in the bloodstream and niche in secondary organs. A zoom on properties, enhanced in EMT-shifted cells, that favor CTC survival in the bloodstream and metastatic niching is depicted. Platelet activation and binding, activation of coagulation: CTCs activate and bind platelets either directly or through molecular intermediates such as fibrin. The liberation of soluble mediators, such as TGF-β from activated platelets, may in return induce/enhance/sustain EMT. In addition, EMT-shifted CTCs express Tissue Factor (TF) that also largely contributes to activate platelets. These coagulation-dependent mechanisms initiate the formation of a fibrin/platelet-rich cocoon around tumor cells that is considered to protect CTCs against shear stress, anoikis, immune attack, and which is determinant for CTC seeding and early niching. Neutrophil recruitment and binding, NETosis: tumor cells secrete soluble factors attracting neutrophils, among other immune cells, and neutrophils in turn secrete EMT-promoting soluble mediators. In addition, neutrophils physically interact with tumor cells and platelets, promoting tumor cell survival and proliferation, and helping CTC arrest on the vascular wall. Furthermore, through their ability to entrap tumor cells in Neutrophil Extracellular Traps, structures also known to favor coagulation events, neutrophils participate to the formation of a protective/anchoring scaffold that supports CTC survival, and facilitate CTC arrest in capillaries and early phases of metastatic niche formation. Immune surveillance: these coagulation/neutrophils-dependent mechanisms of shielding protect CTCs from immune destruction. In addition, CTCs, and more particularly EMT-shifted CTCs, have been reported to harbor increased ability to evade immune surveillance. Among mechanisms involved, the expression of immune checkpoint proteins, such as PD-L1, is likely to enhance their resistance to cytotoxic immune cells. ❸ After an eventual period of dormancy, it is considered that MET processes intervene, favoring metastatic outgrowth. Figure created with BioRender.com.

EMTs thus provide tumor cells with numerous dynamic/reversible properties that help them overcome environmental selective constraints of the metastatic translocation. Here, we will detail crucial mechanisms by which EMTs may impact the fate and metastatic competence of CTCs (Figure 2).

### 4.1. EMT and CTC Release

One of the longest-known properties driven by EMTs is certainly invasiveness. Enhanced invasive ability may be acquired within the primary tumors and contribute to invasion and intravasation, thereby facilitating the release of CTCs in the bloodstream [135,136,137]. EMT-induced proteolytic enzymes, such as matrix metalloproteinases (MMPs), are key players in tumor invasion [138,139], remodeling the extracellular matrix, activating growth factors and weakening intercellular contacts. Although they did not examine EMT markers, Dhar and coworkers supportively reported high MMP activity in CTCs isolated from prostate cancer patients [140]. The ability of EMT to stimulate angiogenesis is also certainly a promoting factor of CTC release. Thus, the expression of the potent angiogenic factor VEGF-A, known as an EMT target gene, has been reported in CTCs of breast cancer patients [141].

Nonetheless, despite the contribution of EMT to CTC release, it is clear today that not all CTCs express canonical EMT markers. Whether EMT characteristics of all EMT^+^ CTC subsets are acquired in the primary tumor or whether some are gained within the circulation are two possibilities that are not mutually exclusive, which may also account for the CTC heterogeneity [68]. Environmental factors present in the bloodstream may indeed also induce/sustain EMT, as detailed in following paragraphs [111,142]. Supportively, single-cell characterization of CTCs taken from different vascular sites in hepatocellular carcinoma (HCC) patients suggests that an EMT-activated phenotype is gained during hematogenous transit [111]. Additionally, a passive mode of entry of tumor cells in the circulation has been suggested and is well in line with the observation of corrupted blood vessels in most tumors [143,144,145]. Such a passive mode of entry may concern single tumor cells but may also liberate clumps/clusters of CTCs in the blood of cancer patients [146,147]. The presence of CTC clumps with metastatic abilities was in fact already recognized in the early 1950s by Watanabe and later by several pioneers in metastasis research [148,149,150]. The mechanisms of cluster formation and their importance in metastasis formation have since then been analyzed more in details using xenografts and experimental metastasis assays performed using a mixed population of tumor cells labeled with different fluorescent reporters [151,152,153]. As an alternative to the passive liberation of clusters in the bloodstream, active collective migration processes of intravasation have also been reported [146,147,152]. Alternatively, an intravascular aggregation of CTCs, particularly at the vicinity of the endothelium, has been identified [153,154], a process that may also involve various hybrid states [155,156].

Overall, whether they are passive or active, EMT-dependent or EMT-independent, these different mechanisms liberate various CTC phenotypes in the bloodstream, and thereby importantly contribute to the heterogeneity of the CTC population.

### 4.2. EMT in CTC Survival and Metastatic Seeding

Once in the circulation, CTCs are subjected to a harsh selective pressure imposed by shear stress, loss of anchorage triggering anoikis, and immune attack [68,157,158]. Studies in mice have accordingly shown that CTCs circulate in the bloodstream for a very short time and that the vast majority of CTCs are rapidly eliminated [159]. Several mechanisms will nevertheless eventually be deployed, enabling few CTCs to survive in the bloodstream and form a niche at secondary sites (Figure 2). Among these mechanisms, those regulated by EMTs are numerous as detailed below.

#### 4.2.1. Activation of Survival Pathways

Some CTCs may activate autonomous survival mechanisms. In this context, an abundant literature shows that EMTs activate many survival pathways (e.g., activation of Akt, PI3K or EGFR pathways, induction of Bcl-2 antagonizing p53 activity), enabling EMT-shifted cells to better resist apoptosis or anoikis [160,161]. This enhanced survival ability of EMT-shifted cells certainly plays crucial role in their now-well recognized resistance to chemo- or targeted therapies [81,162,163,164,165,166]. Supportively, molecular actors of survival pathways (e.g., EGFR, Akt, PI3K) have been detected in CTCs isolated from several types of cancer, sometimes in association with canonical EMT markers such as EMT transcription factors or stem cell markers [101,104,110,167,168,169,170,171,172,173,174] (Table 1).

#### 4.2.2. Traveling in Clusters

Regardless of the mechanisms implicated in the generation of clusters discussed above, traveling as clusters is currently considered to enhance CTC survival and niching at secondary sites. Although much less prevalent than isolated CTCs, experimental data in mouse models show that CTC clusters have higher metastatic potential [146,147,175]. Clusters of CTCs have been detected in a variety of epithelial cancers and, in some studies, their abundance has been associated with poor prognosis as shown in breast, lung, pancreatic, prostate, or kidney cancers [29,109,151,176,177,178,179,180,181,182,183,184]. If their bigger size certainly facilitates their arrest within capillaries, it appears that traveling as clusters may help CTCs survive in the bloodstream [185,186].

If the prevalence of EMT phenotypes in clusters is so far largely uncovered, it is nevertheless recognized that CTC clusters contain hybrid E/M phenotypes. Thus, single-cell RNA sequencing of clustered CTCs versus isolated CTCs from breast cancer patients has revealed that several cell–cell contact molecules are overexpressed/maintained in clusters, such as plakoglobin, which has been further shown to be functionally involved in cluster metastatic potential [151]. Complicating the theory and blurring the border between E and M states even further, EMT/CSC markers may also be implicated in cell–cell interactions in clusters. Accordingly, CD44, which has been consistently reported on CTCs in most types of cancer [50,100,187,188], was recently shown to contribute to intravascular aggregation of tumor cells through the formation of homophilic intercellular interactions [153]. CD44^+^ cell aggregates were also found to be more resistant to apoptosis than single cells. Other recognized markers of mesenchymal or CSC phenotypes have also been detected in CTC clusters in vitro or in mouse models such as Tenascin C or Jagged1 [152,189]. A recent study of CTCs isolated from breast cancer patients and mouse models, reported that the binding sites for CSC transcription factors such as OCT4, SOX2, or NANOG, are hypomethylated in CTC clusters compared with isolated CTCs [49]. Other data collected from human samples also emphasize the presence of hybrid E/M phenotypes in heterogeneous CTC clusters, particularly in lung cancers [51,52,109,190]. Very elegantly, a study by Yu and coworkers identified cells expressing both epithelial (such as EpCAM or cytokeratins) and mesenchymal markers (including fibronectin, N-cadherin or PAI-1) in isolated CTCs but also in CTC clusters from breast cancer patients [179].

Therefore, the presence of EMT-hybrid CTCs in clusters may combine the ability to establish cell–cell interactions, contributing to a better resistance to anoikis/apoptosis, with known pro-metastatic EMT-driven properties (stemness, activation of survival pathways, resistance to apoptosis, enhanced niching properties). This phenomenon may thus contribute to an overall better survival and higher seeding efficiency of clustered tumor cells [191].

Though clusters may be homotypic, they are often heterotypic, containing normal host cells and components, either from the primary tumors [192] or incorporated during their metastatic translocation, as we discuss below.

#### 4.2.3. Interactions with Host Cells and Host Systems

During their metastatic translocation, CTCs continuously and reciprocally interact with host components and cells in all microenvironments encountered. Many of these interactions facilitate both CTC intravascular survival and metastatic niching.

##### Activation of Coagulation

The activation of the coagulation system is today recognized as a crucial process facilitating early metastasis. Hypercoagulability is actually a long-known correlate of malignancy (Trousseau’s syndrome), and venous thromboembolism (VTE) has been associated with worse prognosis [193,194,195]. Accordingly, the CTC count has been associated with hypercoagulability, increased risk of venous thrombosis and dismal prognosis [196,197,198,199,200]. Abundant experimental studies have supportively demonstrated the beneficial effects of anticoagulant strategies in inhibiting metastasis [201]. Many clinical trials examining the impact of the new generation of anticoagulant strategies in cancer are ongoing [202].

If the mechanisms linking cancer and the coagulation system are numerous and multifactorial, platelets are certainly central players [203,204,205,206,207]. Abundant literature indeed demonstrates that tumor cells bind to and activate platelets [203]. Tumor cell/platelet interactions have been shown to engage several receptors including P-selectin or αIIbβ3 on platelets and ανβ3 integrin, PSGL-1 or CD97 on tumor cells [208,209,210,211,212]. Fibrin, the end product of the coagulation cascade, may also bridge tumor cells to platelets. A mechanism by which fibrin connects ICAM-1 on tumor cells to integrin αIIbβ3 on platelets has been particularly highlighted [213]. Interestingly, CD44 has also been described as a receptor that binds P-selectin on platelets, as well as a fibrin receptor [214]. Platelet activation also liberates abundant soluble mediators that may favor the recruitment/activation of host cells such as neutrophils, and may also impact the tumor cell phenotype. All these interactions may thus favor the formation of fibrin/platelets aggregates trapping CTCs and host cells that have also been referred to as circulating tumor microemboli (CTM) [203,204,205,206,207].

The formation of a fibrin/platelets network around tumor cells is considered to favor CTC survival in the bloodstream [203,204,205,206]. It is recognized as an important mechanism shielding and protecting CTCs against shear stress [215] and natural killer (NK) elimination [216,217]. In addition to their contribution to a physical shielding, platelets have also been shown to decrease NK cell antitumor activity through a TGF-β-mediated decrease in NKG2D [218]. A platelet coat around CTCs may also expose platelet MHC-class I molecules and thus “hide” CTCs from NK cells [219]. Biggerstaff and coworkers have precisely shown that soluble fibrin augments platelet/tumor cell interactions in vitro and in vivo [213], hinders cellular cytotoxicity against tumor cells and increases experimental metastasis [220]. In addition, platelet/fibrin-dependent mechanisms also facilitate tumor cell arrest on the vascular wall and their niching at secondary sites [221,222]. If tumor cells possess receptors (CD44, P-selectin, integrins,...) enabling their adherence to the endothelium [223], fibrin or platelets may indeed also mediate and strengthen such interactions [224,225].

A mechanism by which tumor cells may induce coagulation and platelet activation is through their ability to express factors of the coagulation cascade, among which tissue factor (TF) holds a particular place, linking the processes described above to EMT. If TF displays coagulation-independent pro-tumoral signaling functions, it is essentially known as the major cell-associated activator of the coagulation cascade. Its expression by tumor cells, triggering coagulant properties, has been demonstrated to be determinant for CTC survival and seeding [194,195,206,226]. This has been exemplified in numerous animal studies using tumor cells modified for TF expression or TF blocking antibodies, demonstrating that tumor-cell expressed TF is associated with increased abilities to form micrometastases [227,228,229,230]. Further connecting TF-coagulant functions to metastasis, Palumbo and coworkers performed metastasis assays in mice deficient for fibrinogen and prothrombin, and they demonstrated that TF could support metastasis through mechanisms dependent on these distal hemostatic factors [231,232]. Importantly, few studies, including ours, have identified TF as a target gene of EMTs [233,234]. Interestingly, a relationship between TF expression and CSC phenotypes has also been reported [235,236]. The EMT core transcription factors ZEB1 and Snail were delineated to regulate TF expression [234], and recently, vimentin was reported to stabilize TF mRNA [237]. Interestingly, TGF-β, liberated from activated platelets, has been described to trigger EMTs [142], suggesting a regulatory loop between EMT and platelet activation. In addition to these experimental data, the TF/EMT relationship was also evidenced in cancer patients with a correlated expression of vimentin and TF in triple-negative breast cancers (TNBC). A subpopulation of CTC expressing TF and vimentin has also been observed in the blood of metastatic breast cancer patients [234]. Through their ability to express TF, EMT-shifted CTCs would thus be more efficient in activating coagulation and building a protective cocoon.

##### Interaction with Neutrophils

CTCs have been shown to communicate with various cells of the immune system, and interactions with neutrophils seem to be essential. In agreement with data reporting that CTCs are surrounded by white blood cells in Pancreatic Ductal AdenoCarcinoma (PDAC) [238], Szczerba and coworkers recently described that CTCs were found in clusters with neutrophils in invasive breast cancer patients [239]. The abundance of these CTC-neutrophil clusters was further associated with shorter PFS. Corroborating this finding, a higher metastatic potential of these CTC-neutrophil clusters has been demonstrated in experimental metastasis assays.

The mechanisms by which neutrophils may support CTC metastasis are various. Neutrophils have been proposed to escort CTCs and enable cell cycle progression [239]. Along with their ability to secrete soluble factors attracting neutrophils (such as G-CSF, CXCL1, CXCL8, or CXCL5) [240], CTCs indeed directly interact with neutrophils through different receptors including VCAM1 [239], ICAM-1 [241], and β1 integrin [242].

Neutrophils may also interact with CTCs through the coagulation system. Thus, platelets that, as discussed above are inseparable travel companions of CTCs, may bridge neutrophils to tumor cells. The release of soluble mediators (such as CXCL5 and CXCL7) by activated platelets accordingly contributes to neutrophil recruitment [221]. In addition to platelets, fibrin also mediates tumor cell/neutrophil interactions. A sequential binding of αvβ3 and ICAM-1 has been shown to determine fibrin-mediated melanoma adhesion of CD11b/CD18 (Mac-1) to neutrophils [243].

A role for neutrophils in facilitating CTC arrest on the vascular wall has also been evidenced [244,245]. Using an elegant in vitro model consisting of a microfluidic chip covered by HUVEC endothelial cells to mimic the vascular compartment, Chen et al. revealed interactions implicating CD11b on neutrophils and ICAM-1 on cancer and endothelial cells, which favored the formation and arrest on the endothelium wall of tumor cell/neutrophil complexes [244].

Neutrophils may also facilitate CTC survival and niching competencies given their ability to form neutrophil extracellular traps (NETs). These web structures have been identified to capture CTCs, to contribute to a physical protection against shear stress and to facilitate early phases of metastasis (arrest and adhesion on the vascular wall, extravasation and niching) in different mouse cancer models [246,247,248,249,250,251,252]. Conversely, tumor cells may activate neutrophils, thereby stimulating NETosis as shown in vitro and in mouse tumor models [248,253]. Another mechanism by which NETs favor CTC survival and colonization is through their ability to activate coagulation and thrombosis [254,255,256,257,258]. NETs have indeed been shown to activate platelets. In turn, activated platelets promote NETosis [259] and, through the liberation of soluble mediators, further recruit neutrophils to the site. Linking CTCs, neutrophils, platelets and coagulation, NETs may thus contribute to the creation of a protective/anchoring scaffold, helping CTCs to survive in the circulation, arrest in capillaries and niche in secondary sites. Supporting these experimental data, NETs have been associated with a poor prognosis in several cancer types and with cancer-associated VTE [260,261]. Recapitulating these relationships in renal cell carcinoma patients, Wen et al. reported that a high CTC count correlates with elevated levels of fibrinogen and RNA expression of NET’s markers in blood leukocytes [262].

Overall, narrow relationships may thus be established between CTCs, neutrophils, and the coagulation system, with many activation/induction loops existing between the different molecular and cellular actors involved [263]. Although this remains largely uncovered, numerous studies support the idea that EMTs could be central players in such loops and, at least partly, influence or be influenced by most of the above-discussed mechanisms.

Adding to their capacity to induce TF expression, EMTs are indeed known to induce several receptors mediating interactions between neutrophils and CTCs, or between CTCs and platelets/fibrin including CD44 [96,97,99], ICAM-1 [264], αvβ3 [265,266,267], or VCAM1 [268].

Additionally, EMTs importantly modulate the secretome of tumor cells notably by inducing the expression of soluble factors that may act as chemoattractants, or activate the pro-tumoral activities of many inflammatory cells including neutrophils [269,270,271]. Reciprocally, neutrophils have also been shown to induce EMT in several cellular systems [269,270], mostly through the release of soluble factors including CXCL-1 [272], IL-17 [273] or neutrophil elastase [274].

Together, these data suggest that EMT-shifted CTCs would be particularly efficient in deploying coagulation-dependent and stimulating neutrophil-mediated strategies that favor survival, resistance to shear stress and initiation of the metastatic niche.

##### Immune Escape

A crucial property favoring the metastatic potential of CTCs is the ability to escape cytotoxic immune cells. As discussed above, coagulation-dependent mechanisms have been implicated in improved resistance of CTCs to NK-mediated clearance but other mechanisms have also been identified. Although EMT has been reported to stimulate some antitumor immune cytotoxicity [275], literature generally supports an improved resistance of EMT hybrid phenotypes to immune cytotoxic cells. Mechanisms induced by EMT such as increased expression of immune checkpoint proteins, altered autophagy, immunoproteasome deficiency and dysfunction of immunological synapses have been implicated and reviewed elsewhere [276,277]. More particularly in the context of CTCs, a reduced expression of ULBP1 (a major ligand of NKG2D) has been reported in EMT-shifted CTCs isolated from gastric cancer patients (Table 1) and in TGF-β-induced cells in vitro, and a mechanism has been proposed by which EMT-shifted CTC resistance to NK cells is increased [134]. In contrast, López-Soto and coworkers reported an enhanced susceptibility to NK cells and an increased expression of different NKG2D ligands in colorectal cancer cells induced to EMT by several means (TGF-β stimulation, inhibition of glycogen synthase kinase-3β, or Snail overexpression) [278]. The impact of EMT in modulating NKG2D-mediated antitumor response may likely be context-dependent and thus remains to be clarified, particularly in CTCs. Additionally, and together with the advent of immunotherapy, the expression of immune checkpoint proteins such as PD-L1 has been examined and detected on CTCs in different types of cancer [279,280,281]. Supporting an enhanced ability of EMT-shifted tumor phenotypes to better resist immune cytotoxic cells, correlated expression of PD-L1 expression and EMT markers has been evidenced in tumors and CTCs, particularly in NSCLC (non-small cell lung cancer) and TNBC [130,280,282,283,284,285,286,287]. Accordingly, PD-L1 has been identified as a gene regulated by EMTs in various cell systems [284,288,289,290,291,292,293].

These mechanisms of resistance to cytotoxic immune cells could thus contribute to a better survival of EMT^+^ CTCs in the bloodstream but also in the metastatic niche [82].

### 4.3. EMT in the Initiation of the Metastatic Niche

The timing of metastatic niche formation, and the implicated molecular and cellular entities are still poorly understood. It is generally recognized that, after CTC arrest in the vasculature (thus becoming disseminated tumor cells—DTCs), the niche will provide support to help DTCs recover from the stress endured in the bloodstream [82]. In this initiation phase, the niche signals to control EMT/MET plasticity and maintain/accentuate stem cell properties, thereby preventing differentiation, ensuring survival, and facilitating a quiescent state that, if prolonged, may result in dormancy. Whether EMT and CSC features are intertwined or somehow uncouple in this context is still a subject of debate [294]. A single cell analysis study in a breast cancer PDX model thus showed that CTCs/DTCs seeding in secondary organs displayed increased expression of stem cell-, EMT-, prosurvival-, and dormancy-associated genes [295]. Another study in MMTV–Her2 mice reported that a majority of early DTCs express Twist and are in a dormant state [296]. Accumulating data support that EMT-shifted CTCs have an enhanced ability to stimulate niche formation. Accordingly, all mechanisms mentioned in the previous section (enhanced survival properties, coagulation/TF/platelet/fibrin/thrombin-related mechanisms, interactions with neutrophils/NETs) have not only been shown to protect CTCs in the bloodstream and facilitate arrest in the vasculature but have also been demonstrated to contribute to niche initiation. Thus, coagulation/TF [226,297,298,299], platelets and fibrin[ogen] [207,221,222,300,301,302] and NETs [248] are all processes/entities/molecules that have been shown to play crucial roles in early phases of metastatic niche formation. If the microthrombus scaffold engendered by these mechanisms certainly contribute to the formation of an adequate matrix for the DTCs to niche, it also constitutes an environment in which host cells are recruited that are determinant for the consolidation of the niche environment. For instance, Gil-Bernabé and coworkers emphasized that the recruitment of monocytes/macrophages by TF-mediated coagulation is a determinant for tumor cell survival and metastatic niche establishment in mouse models [303]. Platelet-dependent processes also strongly support the recruitment of inflammatory cells into the niche [207,221,222,300,301,302]. It is also plausible that EMT hybrids are particularly efficient at establishing the immunosuppressive environment observed during niche initiation [304]. Although this was not studied in the particular context of niche formation, EMT-shifted cells have thus been shown to recruit immunosuppressive populations of immune cells [305,306,307] through the secretion of immunosuppressive mediators (e.g., CCL2, CXCL8, thrombospondin). The ability of EMT hybrids to better resist cytotoxic immune cells is also certainly a property facilitating the establishment of the metastatic niche.

Additionally, once arrested in the vasculature of colonized organs, EMT^+^ DTCs may also establish privileged interactions with resident host cells to initiate niche activation. In a mouse metastasis model, del Pozo et al. thus showed that Axl expressed by mesenchymally-shifted metastatic initiating cells is involved in the niche activation through the regulation of thrombospondin 2 secretion and the education of resident fibroblasts [308]. Subsequently, these DTCs reverted to an Axl-negative, more epithelial phenotype to proliferate. The ability of mesenchymally-shifted CTCs to accomplish early niching and a subsequent reversion to a more epithelial phenotype associated with metastatic outgrowth has also been observed by other authors [309,310]. Thus, in later stages of metastasis, the niche signals to induce MET and favor proliferation leading to metastatic outgrowth.

All in all, these abundant experimental data, considered together with the molecular characterization of human CTCs, support that mesenchymally-shifted hybrid CTCs may represent subpopulations with enhanced competence to survive in the circulation and to initiate niche formation at distant sites. If many mechanisms detailed above (activation of coagulation, interactions with neutrophils) are likely to favor the formation/consolidation of heterotypic clusters/CTM, they may also help isolated CTCs to overcome the constraints of metastatic translocation and niching. Various hybrid phenotypes may therefore travel and niche as isolated CTCs while others, potentially more dependent on homo/heterotypic interactions for survival, may travel and nest as clusters, thereby gaining even increased metastatic competence. Considering experimental data and clinical observations showing that isolated CTCs are largely predominant entities, it is very likely that these two scenarios coexist.

## 5. Functional Assays for CTCs

Current CTC research invests great efforts in the development of ex vivo, in vitro and in vivo models allowing a functional characterization of CTCs [311]. An underlying aim is certainly to understand CTC biology and to identify/isolate, within a heterogeneous population of CTCs, those CTCs with a high potential to initiate metastasis (so-called MICs, metastasis initiating cells).

Because CTCs are so rare, many researchers have aimed at shortly expanding CTCs in culture and even at establishing CTC-derived cell lines before downstream in vitro and in vivo characterization [312,313], although this may certainly modify the heterogeneity of the initial CTC population and introduce a bias. Cell lines have thus been successfully established from different CTC subpopulations isolated from different types of cancer including breast [314,315], colon [316], and lung [126,317,318] cancers. To better mimic the in vivo contexts, 3D (tumor) spheroids and organoids culture models are being optimized [319,320,321,322,323,324,325,326,327]. Additionally, 3D co-culture systems are also under development [317]. Although this remains largely uncovered, several studies have examined EMT heterogeneity in such culture models aiming to gain knowledge about CTC biology and their metastasis-initiating potential. Hybrid E/M phenotypes have thus been reported in CTC-derived cell lines [126,316]. Dynamic differences in E and M composition have also been observed in a 3D polymer scaffold culture [323] and in 3D spheroid cultures [324]. Aiming at isolating and characterizing invasive CTCs, Vitatex^TM^ developed a platform system to capture and culture viable CTCs with an ability to adhere and remodel/ingest a labeled matrix (Cell Adhesion Matrix -CAM) [187,328,329]. Friedlander and coworkers were thus able to identify invasive CTCs expressing EMT/CSC markers (vimentin, CD44) in the blood of prostate cancer patients [187]. Most importantly, in vivo CTC xenografts (CTC-derived xenografts, CDX) are also being optimized as most representative models to evaluate the metastatic competence of different subpopulations of CTCs and understand the biology of MICs. Although some studies use CTCs without prior in vitro expansion, CTC-derived cultures or cell lines are also commonly assessed. MICs have thus been successfully identified in CTCs isolated from different types of cancer including breast, lung or colon cancer [315,316,330,331,332,333,334]. In some studies, intravenous or intra-femoral injections have been directly performed. Therefore, breast-cancer derived CTC cell lines expressing E/M hybrid phenotypes (cytokeratins 8/18, vimentin, CD44) have been shown to metastasize after tail-vein or intracardiac injection [314]. Similar findings have been reported with CTC cell lines established from an initial xenograft model of a breast cancer-derived DTC cell line [334]. Baccelli and coworkers also identified CD44^+^/c-Met^+^/CD47^+^ CTCs isolated from breast cancer patients, which generated bone, lung, and liver metastases after intrafemoral injection [335].

Another important scope behind the elaboration of these CTC-derived assays is to develop patient-matched preclinical models that could be established for longitudinal assessment of disease progression and drug sensitivity, thereby customizing and improving individual patient management [320,321]. Thus, CTC cell lines [315,336,337,338] and 3D CTC-derived spheroid and organoids models [317,322,323,324,325,339] have been examined in drug screening settings. In this sector, microfluidics is also making a breakthrough, allowing a better control of the culture conditions and easier settings for drug delivery [340]. CDX have also been evaluated in drug screening assays [341,342,343] with an ultimate goal of being analyzed in patient-matched settings [344]. Although this line of research is very promising, further developments are needed to improve efficiency and reproducibility and, ultimately, to select models that could be transferrable in co-clinical trials on patients and patient-matched “avatars”.

## 6. Clinical Relevance of EMT-Related CTC Heterogeneity

A profusion of clinical studies supports the validity of CTC count before or during treatment (chemotherapy or targeted therapy) as a prognostic biomarker particularly for breast, prostate, and colorectal cancer [7,8,345,346,347,348,349,350]. Nevertheless, CTCs are thus far not utilized in clinical routine. Large-scale multicentered trials using identical detection methods seem crucial to establish the utility of CTC enumeration and implement this parameter in clinical practice.

In addition to enumeration, it is also becoming clear that a deeper molecular characterization of CTCs may provide a goldmine of information for clinicians and holds promises to improve personalized cancer management.

### 6.1. EMT^+^ CTCs as A Prognostic Factor

Numerous studies to date have already suggested that certain EMT-shifted CTC subsets harbor prognostic information. In agreement with the well-documented and numerous pro-metastatic functions of EMTs, the detection of certain EMT actors in CTCs has thus been correlated with poor clinical parameters such as an aggressive cancer types and a shortened OS or PFS (Table 1). More particularly, CTCs harboring mesenchymal features have been reported to associate with the presence of metastases in numerous cancers including breast, lung, pancreatic, colorectal, prostate, or hepatocellular cancers [101,109,114,116,122,125,169,351,352,353,354]. Considering particular EMT/CSC molecular actors, studies, for instance, have detected CD44, often in conjunction with other canonical EMT markers, in CTCs isolated from many cancer types, which was associated with poor clinical outcomes in some studies [50,100,170,187,188,335,355,356]. EMT transcription factors (Twist, ZEB or Snail) have also been frequently detected in CTCs and associated with a poor prognosis in different cancer types [101,174,353,357,358]. Axl was found in CTCs isolated from lung cancers, particularly in those expressing vimentin [52], and it was similarly associated with a poor prognosis [132]. Interestingly, high expression of PD-L1 and EMT markers in CTCs was reported to be a sign of a grim prognosis in patients with complete surgically resected lung cancer [130]. The exploration of EMT on CTCs may thus help to predict a poor outcome and could thus guide towards an adaptation of individual patient management (reinforced surveillance, treatment adaptation).

### 6.2. EMT^+^ CTCs in Therapy Management

In addition to this important prognostic information, EMT detection in CTCs also harbors meaningful predictive information. Identifying molecules that may predict the response or a non-response to an existing treatment accordingly constitutes a promising perspective of CTC molecular characterization that may guide/refine patient stratification and management, particularly when tumor biopsy is not informative or no longer an option.

Therefore, a combined detection of known therapeutic targets (such as EGFR, PD-L1 or HER2) together with poor-prognostic EMT markers may refine patient management and point to potential combinatory therapies. Illustrating this, an association between PD-L1 expression and EMT markers has been evidenced in tumors and CTCs, particularly in NSCLC and TNBC [130,280,282,283,284,285]. Along these lines, tandem expression of vimentin and PD-L1 has been shown to constitute a prognostic factor in NSCLC [287]. Hence, EMT has been proposed as a candidate biomarker to be explored on tumors and CTCs to predict immunotherapy outcome and to design combination approaches with immunotherapy [276,359].

A pivotal aspect of CTC analysis resides on the possibility to perform successive liquid biopsies to monitor the progression of the disease and to assess treatment efficacy. Importantly, the emergence of EMT-shifted CTC phenotypes after one or several lines of treatment was found to correlate with drug resistance in several studies [52,104,112,121,124,179,360]. This finding is in agreement with a large amount of experimental data demonstrating an enhanced ability of EMT-shifted cells to resist most existing therapeutic options (chemo- radio-resistance, and resistance to existing targeted therapies) [81,162,163,164,165,166]. Analyzing EMT as a companion marker in the course of a treatment may thus help predict drug resistance and eventually help guide therapeutic strategies.

In addition, a deeper molecular characterization of these drug-resistant phenotypes may also point to the emergence of other targetable pathways, or identify new potential therapeutic targets. For instance, NSCLC patients treated with one or several lines of therapies, progressively presented significantly more vimentin-positive Axl-expressing CTCs [52]. Overexpression of Axl in CTCs isolated from lung cancer patients who developed resistance to EGFR inhibitor therapies has also been reported [361]. Examining Axl on CTCs, against which drugs are currently evaluated in clinical trials, has accordingly gained interest within the last 5 years [132,362,363].

As debated numerous times [75,364], the dynamic and reversible nature of EMTs makes it difficult to define a single EMT molecular signature that fits the variety of hybrid phenotypes, particularly across various types of cancer. Due to this molecular complexity, the identification of specific EMT/CSC molecular markers or signatures to be used as prognostic or predictive markers in specific cancer contexts will most likely constitute a necessary steppingstone towards a clinical use. Defining an “EMT index” to somehow quantify the extend of EMT may also facilitate the implementation of EMT analysis in clinical practice. Fici and coworkers, analyzing EMT-related splicing factors ESRP1/ESRP2/RBFOX2, provided rationale to use the ESRP1/RBFOX2 ratio as a prognostic biomarker for early prediction of metastasis in breast cancer, and further suggested this ratio could also be evaluated in CTCs [365]

Further development is thus ongoing to delineate clear parameters (selection of appropriate CTC isolation techniques, selection of specific EMT markers or EMT signatures or EMT indexes, selection of adequate preclinical models for drug screening) that may be transferable to clinical routine. With increasing experimental data identifying new EMT molecular mechanisms supporting metastatic competence, emerging therapeutic targets will also undoubtedly be identified. Thus, targeting mechanisms and specific molecules involved in coagulation, NET formation or neutrophil interactions are currently interesting paths of exploration.

## 7. Concluding Remarks

A tremendous amount of experimental data shows that EMTs endow epithelial tumor cells with properties that help them overcome hostile signals encountered as they travel as CTCs: an enhanced survival potential, an increased ability to activate coagulation, an augmented aptitude to hijack host cell pro-metastatic functions, and a raised capacity to establish an early metastatic niche.

Nevertheless, examining EMT in human CTCs remains complicated, partly because enrichment and isolation of rare human CTCs remain a technical challenge. Microfluidic-based approaches of CTC isolation are undergoing rapid development and currently represent the most promising innovative avenue to isolate label-free human CTCs, hopefully accelerating CTC research and subsequently CTC consideration in the clinic.

The clinical potential of CTCs is indeed enormous and multiple. CTCs hold a prominent place in personalized medicine. When tumor biopsy is not or no longer an option, CTCs represent a unique accessibility to tumor material, and, moreover, a material containing the physical entities responsible for metastasis. Additionally, as liquid biopsies are non-invasive procedures that can be repeated during the course of the treatment, a live assessment of disease progression and a monitoring of treatment efficacy may be possible.

Thus far, and beyond the clinical interest of CTC enumeration, data collected on human CTCs point to a clinical utility to longitudinally interrogate the EMT status in CTCs. An EMT signature in CTCs is indeed a marker of poor prognosis in most cancer contexts. In advanced and metastatic situations, EMT traits also clearly point to drug resistance. EMT detection may thus represent a companion marker of further assessment to facilitate individual cancer patient management and guide treatment decisions. Although the examination of specific EMT markers seems easier to implement in the clinic, analysis of EMT signatures may harbor more significant power and point to new potential therapeutic targets to consider.

Additionally, the elaboration of preclinical models that would allow a deeper functional and molecular characterization of MICs within the CTC population and that could be utilized as drug-screening platforms, certainly represent a crucial axis of CTC research that may pave a way towards personalized medicine.

## Figures and Tables

**Table 1:** Detection of epithelial–mesenchymal transition (EMT) and stem-cell markers in CTCs from cancer patients.

EMT and Stemness Markers (+Other Associated)	Type of Tumor	Method of Separation/Characterization	Method of Detection	No. of Patients	Correlation between EMT Markers and Clinical Parameters	Ref.
EpCAM, CK8, CK19, CDH1, Vimentin, CDH2, ZEB1, CD24, CD44, ALDH1, MCAM, SPARC	Pancreatic Cancer	Lymphoprep™ + MINDEC	IF and single-cell multiplex RT-qPCR	21	Presence of M^+^ CTCs and E^+^ CTCs	[100]
EpCAM, CK8, CK18, CK19, Vimentin, Twist	Colorectal Cancer	CanPatrol system™	RNA-FISH	1203	CTC count and M^+^ CTCs correlate with clinical stages, and lymph node and distant metastasis	[101]
CK19, Twist, Snail1, Snail2, ZEB1, FOXC2, tPA	Breast Cancer	RosetteSep™	RT-qPCR, ELISA (tPA)	110	- Presence of E^+^ and M^+^ CTCs. - No higher tPA levels in CTCs compared with healthy donors	[102]
EpCAM, CK8, CK18, CK19, Vimentin, Twist, OCT4	Lung Cancer	CanPatrol system™	RNA-FISH	37	M^+^ CTCs associated with distant metastasis and correlated with high total CTC counts	[103]
EpCAM, Muc-1, PI3Kα, AKT-2, Twist	Ovarian Cancer	AdnaTest (OvarianCancer Detect + EMT Detect)	RT-qPCR	95	- EMT^+^ CTCs increase after chemotherapy - PI3Kα^+^ EMT^+^ CTCs in combination with E^+^ CTCs have a poor prognosis	[104]
EpCAM, Vimentin	Lung Cancer	TelomeScan F35	IF	123	EMT^+^ CTCs: poor response to chemotherapy and correlation with a decreased PFS	[105]
EpCAM, Vimentin, CK8, CK18, CK19, E-Cadherin, ZEB1, Snail1	Prostate Cancer	- CellSearch^®^, RosetteSep™ - EasySep™ + Anti-vimentin selection	IF	48	ND	[106]
CK, Vimentin, C-MYC, PTEN	Prostate Cancer	Parsortix™	FISH	81	- CK^−^ Vimentin^+^ CD45^−^ CTCs correlated with disease burden, tumor aggressiveness, and poorer survival - CK^+^ Vimentin^+^ CD45^−^ CTCs: associated with metastasis	[48]
EpCAM, CK8, CK18, CK19, Vimentin, EGFR, KRAS	Lung Cancer	CellSearch^®^	IF	125	C - CTCs > 5: reduced OS - No difference between Vimentin^+^ CTCs and Vimentin^-^ CTCs regarding OS - Increased Vimentin^+^ CTCs in patients with EGFR mutations	[107]
EpCAM, CK19, MUC1, HER2, FLT1, EGFR, GZMM, PGR, CD24, KIT, PLAU, ALDH1, CTSD, MKI67, Twist,	Metastatic Breast Cancer	AdnaTest (EMT2/StemCellSelect™)	46 gene-signature qPCR	45	-14 genes significantly higher (CK19, ALDH1, EGFR, EpCAM, Twist) in CTC-harboring patients versus CTC-negative patients -ADAM17 expressed in CTCs from treatment-resistant patients	[108]
EpCAM, CK8, CK18, CK19, Vimentin, Twist	Colorectal Cancer	CanPatrol system™	RNA-FISH	126	M+ CTCs and CTM associated with tumor metastasis	[109]
EpCAM, CK20, Survivin, PI3Kα, AKT-2, Twist, ALDH1	Metastatic Colorectal Cancer	Dynabeads^®^	RT-qPCR	78	- AKT-2^+^ CTCs: shorter median PFS - ALDH1^+^ CTCs: shorter OS - ALDH1^+^, PIK3Kα^+^ and/or AKT2^+^ CTCs: shorter PFS and OS	[110]
EpCAM, CK8, CK18, CK18, Smad2, β-catenin	Hepatocellular Carcinoma	CellSearch^®^	Single-cell RNA analysis	73	- CTCs and CTC cluster count correlated with poor prognosis - CTCs seems to be predominantly epithelial at release and switch to EMT phenotype during hematogenous transit	[111]
CK19, EpCAM, CDH1, HMBS, PSCA, ALDH1, CD133, HPRT1, Twist, Vimentin, N-cadherin, B2M, PLS3, PSA	Prostate Cancer	CellSearch^®^, PSA EPISPOT and CellCollector^®^	IF/RNA	108	EMT markers increased after radiotherapy	[112]
EpCAM, CK8, CK18, CK19, Vimentin, Twist	Hepatocellular Carcinoma	CanPatrol system™	RNA-FISH	62	M^+^ CTCs associate with higher recurrence after curative resection	[113]
EpCAM, CK8, CK18, CK19, Vimentin, Twist, β-catenin	Hepatocellular Carcinoma	CanPatrol system™	RNA-FISH	112	A large number of M^+^ CTCs associated with early recurrence, multi-intrahepatic recurrence, and lung metastasis.	[114]
EpCAM, CK8, CK18, CK19, Vimentin, Twist	Hepatocellular Carcinoma	CanPatrol system™	RNA-FISH	165	M^+^ CTCs correlated with high AFP levels, multiple tumors, advanced TNM and BCLC stage, presence of embolus and shorter RFS	[115]
EpCAM, CK8, CK18, CK19, Vimentin, Twist, LGR5	Colorectal Cancer Prognosis	CanPatrol system™	RNA-FISH	66	M^+^ CTCs correlated with advanced stages, metastasis, and shorter PFS and OS	[116]
EpCAM, CK8, CK18, CK19, Vimentin, Twist, Glypican 3	Hepatocellular Carcinoma	CanPatrol system™	RNA-FISH	80	Twist^+^ CTCs correlated with poor clinical outcome (portal vein tumor thrombi, TNM stages, cirrhosis, tumor number, tumor size)	[117]
EpCAM, CK8, CK18, CK19, Vimentin, Twist	Esophageal Squamous Carcinoma	CanPatrol system™	RNA-FISH	21	Number of CTCs correlate with E/M^+^ CTCs and M^+^ CTCs	[118]
EpCAM, CK8, CK18, CK19	Prostate Cancer	CellSearch^®^ + CellSieve Microfilter™	IF	108	- EpCAM^high^ CTC>5 shorter OS - No correlation with EpCAM^low^	[119]
EpCAM, HER2, EGFR	Breast Cancer	Parsortix™	IF	43	Hypomethylation of binding sites for OCT4, SOX2 or NANOG are associated with a poor prognosis	[49]
EpCAM, CKs, CD133	Pancreatic Ductal Adenocarcinoma	The Gem device	IF	24	Fluctuation of EpCAM expression in CSC^+^ CTCs	[28]
EpCAM, Vimentin, ALDH1, PALB2, MYC	Metastatic Breast Cancer	- CellSearch^®^ (enumeration) - Lymphoprep™ (molecular analysis)	RT-qPCR	20	EpCAM^high^, Vimentin^low^, and ALDH1^high^ are associated with shorter OS and PFS.	[120]
CD44	Hepatocellular Carcinoma	The Labyrinth device	IF	16	- Correlation of cluster of CTCs with advanced stages. - 71.4% of single CTCs express CD44	[50]
CK8, CK18, CK19, ALDH1, Twist	Metastatic Breast Cancer	Ficoll-Hypaque™	IF	130	- CSC^+^/partial EMT^+^ CTCs correlate with lung metastasis and a decrease in PFS - In HER2^-^ cancers, CSC^+^/partial EMT^+^ CTCs correlate with a reduction of OS	[121]
EpCAM, CK8, CK18, CK19, Twist, Vimentin	Lung Cancer	CanPatrol system™	RNA-FISH	110	M^+^ CTCs are associated with metastasis	[122]
EpCAM, CK8, CK18, CK19, Twist, Vimentin	Castration Sensitive Prostate Cancer	CanPatrol system™	RNA-FISH	108	Faster disease progression in patients with M^+^ CTCs	[123]
EpCAM, CK8, CK18, CK19, Twist, Vimentin	Breast Cancer (HER2^-^)	CanPatrol system™	RNA-FISH	108	M^+^ CTCs are associated with a poor prognosis	[124]
EpCAM, CK8, CK18, CK19, Twist, Vimentin	Pancreatic Ductal Adenocarcinoma	CanPatrol system™	RNA-FISH	107	M^+^ CTCs are correlated with TNM stage and distant metastasis	[125]
EpCAM, EGFR, CK7	Lung Cancer	Herringbone-Chip	IF	109	EpCAM^+^ EGFR^+^ CK^+^ CD45^−^ CTCs show a negative correlation with clinical stage	[126]
ADAM23, Snail1, Slug, ZEB1, Twist, CK19	Breast Cancer	RosetteSep™	RT-qPCR	203	- Patients with M^+^ CTCs and no ADAM23 hypermethylation have longer DFS than patients with M^+^ CTCs and ADAM23 hypermethylation - Patients with M^+^ CTCs and Ki-67 ^low^ have longer DFS than patients with M^+^ CTCs and Ki-67 ^high^	[127]
Vimentin, TGFβ-RI, MMP2	Metastatic Pancreatic Cancer	ISET^®^ filter	IF	21	No significant differences between the number of CTCs and PFS or OS	[128]
MRP2, MRP5, MRP7, ALDH1, Twist, Snail1	Head and Neck Cancer	RosetteSep™ + Cultured cells	IF	20	High expression of multidrug resistance genes, EMT genes or stem cells markers are associated with a poor PFS	[129]
CK8, CK18, CK19, EpCAM, Vimentin, N-Cadherin, PD-L1	Lung Cancer	CellSieve Microfilter™	IF	30	High levels of PD-L1 and EMT markers in primary tumors and CTCs are associated with poorer survival	[130]
EpCAM, CK8, Vimentin, Twist	Cervical Cancer	CanPatrol system™ + CellSearch^®^	RNA-FISH	90	M^+^ CTCs are associated with malignant stages	[131]
CK8, CK18, CK19, AXL, EGFR	Lung Cancer	Carcinoma Cell Enrichment and Detection Kit (Miltenyi Biotec.)	IF	47 ADC in comparison to 50 SSC	- Poor prognosis of AXL^+^ CTC - EMT^+^ CTCs are correlated with high expression of AXL - EMT^+^ CTCs are associated with high N stages patients - EMT^+^ CTCs are associated with an increased risk of RFS and OS - CTCs after 6 months of surgery: poor prognostic factors for OS	[132]
PD-L1, EpCAM, CK8, CK18, CK19, Vimentin, Twist, PD-L1 EGFR, KRAS, BRAF, ROS1 mutation, ALK rearrangement	Lung Cancer	CanPatrol system™	RNA-FISH	114	Poor prognosis with a high number of CTCs, CTCs with mesenchymal features and PD-L1^+^ CTC	[133]
Vimentin, CK, AXL	Lung Cancer	Microcavity array system	IF	20	High expression of AXL in Vimentin^+^ CTCs - Clusters of CTCs with Vimentin expression	[52]
EpCAM, Vimentin, ULBP1	Gastric cancer	Cyttel-CTC	FISH	41	EMT^+^ CTCs with diminished expression of ULBP1	[134]
EpCAM, Vimentin, CK	Lung Cancer	The Labyrinth device	FISH	25	- Most of CTC clusters are EpCAM^-^ - Poorer PFS for patients with CTC clusters > single CTCs compared with patients with CTC clusters < single CTCs	[51]

Studies published after 2016 are listed in the Table. For earlier studies, please refer to Table 1 in Francart et al. [68]. It is noteworthy that the CanPatrol system based on filtration followed by characterization using cocktails of probes discriminating E and M phenotypes have been extensively used within these last 5 years. ADC: adenocarcinoma; BCLC: Barcelona Clinic Liver Cancer; CKs: cytokeratins; DFS: disease-free survival; IF: immunofluorescence; M^+^ CTCs: mesenchymal circulating tumor cells; ND: not determined; No.: number, OS: overall survival; PFS: progression-free survival; RNA-FISH: RNA fluorescence in situ hybridization; (q)RT PCR: (quantitative) reverse transcription polymerase chain reaction; RFS: relapse-free survival; tPA: tissue plasminogen activator; TNM stage: Tumor Node Metastasis stage.
